# Ultrasonic application to boost hydroxyl radical formation during Fenton oxidation and release organic matter from sludge

**DOI:** 10.1038/srep11419

**Published:** 2015-06-12

**Authors:** Changxiu Gong, Jianguo Jiang, De’an Li, Sicong Tian

**Affiliations:** 1 School of Environment, Tsinghua University, Beijing 100084, China; 2 Key Laboratory for Solid Waste Management and Environment Safety, Ministry of Education of China, China; 3 Collaborative Innovation Center for Regional Environmental Quality, Tsinghua University, Beijing, China

## Abstract

We examined the effects of ultrasound and Fenton reagent on ultrasonic coupling Fenton oxidation (U+F) pre-treatment processes for the disintegration of wastewater treatment plant sludge. The results demonstrated that U+F treatment could significantly increase soluble chemical oxygen demand (SCOD), total organic carbon (TOC), and extracellular polymeric substances (EPS) concentrations in sludge supernatant. This method was more effective than ultrasonic (U) or Fenton oxidation (F) treatment alone. U+F treatment increased the release of SCOD by 2.1- and 1.4-fold compared with U and F alone, respectively. U+F treatment increased the release of EPS by 1.2-fold compared with U alone. After U+F treatment, sludge showed a considerably finer particle size and looser microstructure based on fluorescence microscopy, and the concentration of hydroxyl radicals (OH•) increased from 0.26 mM by F treatment to 0.43 mM by U+F treatment based on fluorescence spectrophotometer. This demonstrated that U+F treatment improves the release of organic matter from sludge.

Sewage sludge is an inevitable by-product of wastewater treatment processes. The total amount of domestic wastewater discharged in 2012 was 6.8 × 10^10^ tons, of which household wastewater contributed 4.6 × 10^10^ tons^1^. Given that the average solid-containing rate of municipal sewage is 0.02%, sludge yield reached 6.8 × 10^7^ tons (moisture: 80%). Large amounts of activated sludge without proper disposal poses a threat to ecological systems^2^. On the other hand, sludge is a potential resource because it contains large quantities of organic matter that can be converted into biogas through anaerobic digestion^3^,^4^. Sewage sludge contains considerable amounts of nutrients^5^,^6^,^7^, especially phosphorus (0.5–0.7% total solid [TS]) and nitrogen (2.4–5.0% [TS])^8^. These nutrients exist mainly in proteinaceous and zoogloea forms^6^. To recover nutrients such as nitrogen (N) and phosphorus (P) from sewage sludge, it is necessary to perform N and P-solubilization processes to release phosphate into the supernatant^9^. To recover nutrients, the structure of sludge zoogloea must be broken down. Biopolymers are released following destruction of the cell structure. Various sludge disintegration methods have been investigated for pre-treatment; these methods disrupt cell walls, resulting in sludge cell lysis^10^. Possible pre-treatments include mechanical, thermal, chemical and biological methods. Recently, it has been reported that ultrasonic pre-treatment could overcome the rate-limiting step and reduce the digestion time^11^. Organic matter was transferred from sludge flocs to the aqueous phase after ultrasonic pre-treatment, which improved the digestibility and dewaterability^10^. Fenton oxidation is commonly used to degrade organics and kill microorganisms during wastewater treatment. During Fenton oxidation, the sludge structure is destroyed and water and organics are released^12^. This reagent also oxidizes odorous substances, kills pathogenic bacteria and stabilizes the sludge^12^,^13^,^14^. Problems such as a relatively low efficiency and high energy consumption are observed using a single disintegration method, while some studies have demonstrated that the combination of various methods with different disintegration modes improves the disintegration effect in a synergistic manner. Kim^15^ and Jin^16^ evaluated alkali coupled with ultrasound as a pre-treatment for sludge disintegration. Kim^15^ reported that alkali could reduce the energy consumption of the ultrasound method. Jin^16^ found that the application order of alkali and ultrasound treatment had a significant effect on the release of sludge chemical oxygen demand (COD). Xu^17^ examined sludge pre-treatment using ultrasound coupled with ozone and showed that ultrasound improved the oxidative ability of ozone and significantly improved its utilization ratio. Many disintegration methods are available, each of which has advantages and disadvantages. Single Fenton oxidation and ultrasound are most commonly used for wastewater treatment^13^,^18^. Some studies have been reported on the degradation of bisphenol A in waste water sludge by ultrasonication, Fenton’s oxidation or combination of both (ferro-sonication) pre-treatment processes^18^,^19^. Ultrasound coupled with Fenton oxidation pre-treatment of sludge was also investigated to enhance the efficiency of sludge reduction and effectiveness of operating cost^20^. Herein, we use ultrasound coupled with Fenton oxidation pre-treatment of sludge to promote sludge disintegration. This technique combines the advantages of mass transfer and cavitation effects of ultrasound and the strong oxidation function of Fenton reagent, resulting in improved sludge disintegration. By examining COD, TOC and EPS release after U+F treatment, the mechanism of sludge disruption was investigated and discussed.

## Material and Methods

### Excess sludge

Excess sludge was obtained from the Xiaojiahe Wastewater Treatment Plant in Beijing, China, which has a sewage treatment capacity of 40,000 m^3^ per day. The sewage treatment plant uses the anaerobic-anoxic-oxic (A2/O) processing system for municipal wastewater treatment, which is the most common method of treating sewage in Chinese cities. Excess sludge was obtained from the secondary sedimentation tank, in which gravity thickening and aerobic digestion occur.

### Experimental set-up

A probe-type ultrasound generator was used in this report. Ultrasound (20 kHz) was used according to previous reports^21^,^22^,^23^.

#### Fenton reagent preparation

As reported in our previous study^21^, the optimal dose of Fenton reagent for sludge disruption was prepared as follows: 2.0 g of FeSO_4_.7H_2_O were added to 1 L of sludge and stirred uniformly, after which 1.7 mL of 30% H_2_O_2_ was added to the system, in which the concentrations of Fe^2+^ and H_2_O_2_ were 0.4 and 0.50 g/L, respectively. A hydrochloric acid solution (18 wt%) was used to adjust the pH to 3 according to the previous study. To investigate the effect of processing time on the release of organic matter, the ultrasonic energy density was fixed at 720 W/L. To investigate the effect of ultrasonic energy density on the release of organic matter, the ultrasonication duration was 20 min. For U+F, Fenton oxidation time was fixed at 20 min, followed by ultrasonication. The settings used for hydroxyl radical assays were as follows: ultrasonic energy density of 720 W/L, ultrasonication time of 20 min for U; Fe^2+^ concentration of 0.4 g/L, H_2_O_2_ concentration of 0.50 g/L, oxidation time of 20 min for F; Fe^2+^ concentration of 0.4 g/L, and H_2_O_2_ concentration of 0.50 g/L for U+F. For EPS extraction from sludge, the sludge samples were filtered using qualitative filter paper, after which the supernatant was centrifuged (6000 rpm for 20 min). Then the sediment was mixed with 1 M NaOH and stirred for 3 h at 4 °C, followed by centrifugation (4000 rpm for 5 min). The liquid supernatant was filtered using a cellulose acetate fiber membrane (0.2 μm) and dried using the filter liquor (100 °C, 24 h).

OH• concentration was detected using a fluorescence spectrophotometer^24^. The OH• concentration was trapped by terephthalic acid (TA). The fast reaction between non-fluorescent TA and OH• produces fluorescent hydroxyterephthalic acid (HTA). Than the concentration of HTA can be determined based on fluorescence measurements and taken as a relative measurement of OH• produced in the solution. It was an indirect method. The concentration of terephthalic acid (TA) was adjusted to 2 mM in sludge samples and then the samples was exposed to U, F, or U+F treatment. The amount of OH• in the supernatant was measured after centrifugation (8000 rpm for 10 min). In addition, we also adopted another methods to detect OH•. The OH• was trapped by 5,5-dimethyl-1-pyrroline-N-oxide and detected with an ESR Spectrometer^25^ as shown in supplementary information file.

### Analytical methods

**Figure 1:**
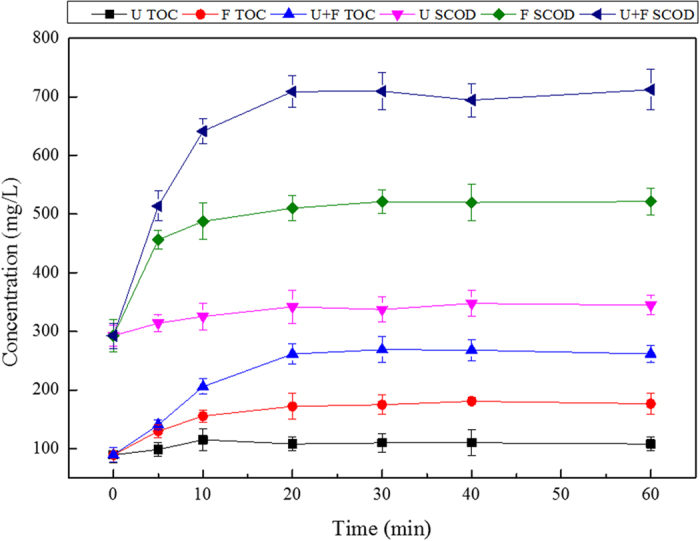
Effects of time on SCOD and TOC released from sludge (ultrasonic energy density of 720 W/L, Fe^2+^ concentration of 0.4 g/L, and H_2_O_2_ concentration of 0.5 g/L).

**Figure 2:**
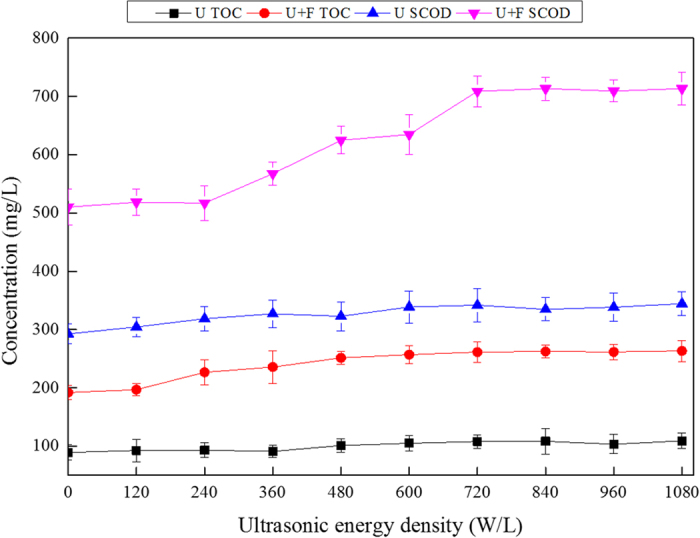
Effects of ultrasonic energy density on SCOD (**a**) and TOC (**b**) release from sludge (treatment time of 20 min; Fe^2+^ concentration of 0.4 g/L, H_2_O_2_ concentration of 0.5 g/L).

**Figure 3:**
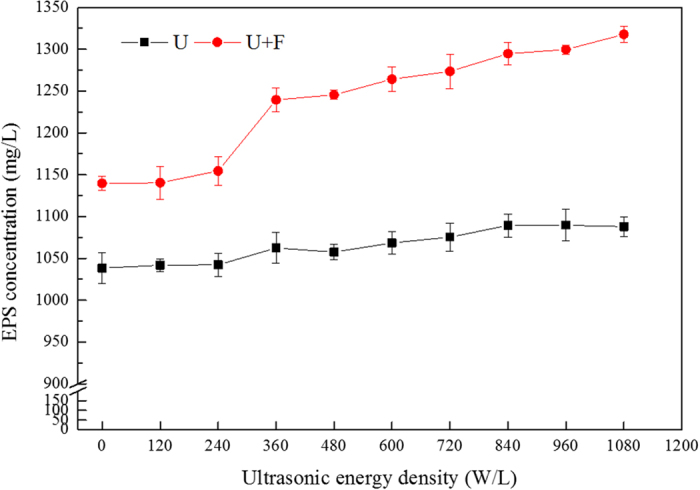
Effects of ultrasonic energy density on EPS release from sludge (treatment time of 20 min, Fe^2+^ concentration of 0.4 g/L, H_2_O_2_ concentration of 0.5 g/L).

**Figure 4:**
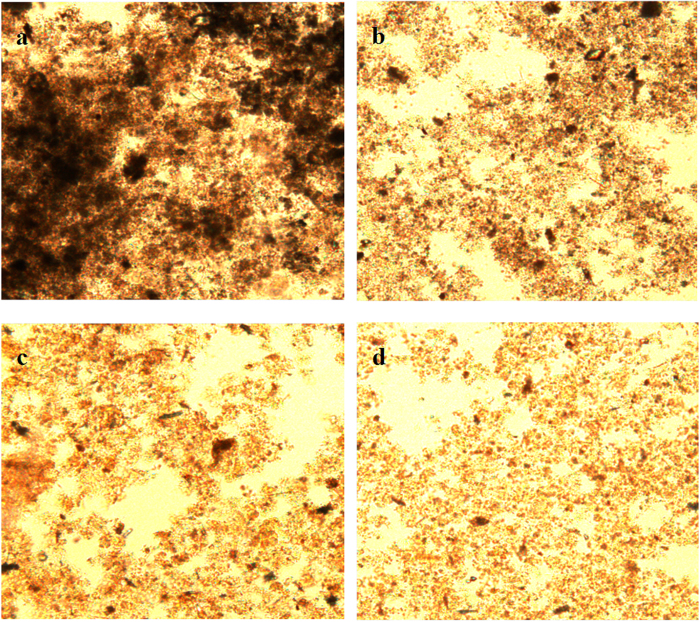
Fluorescence microscopy images of sludge after the U, F and U+F treatments (**a**: raw sludge; **b**: sludge treated with U for 20 min at an ultrasonic energy density of 720 W/L; c: sludge treated with F for 20 min at Fe^2+^ concentration of 0.4 g/L and H_2_O_2_ 0.5 g/L; d: sludge treated with U+F for 20 min at an ultrasonic energy density of 720 W/L with Fe^2+^ concentration of 0.4 g/L and H_2_O_2_ of 0.5 g/L.)

**Figure 5:**
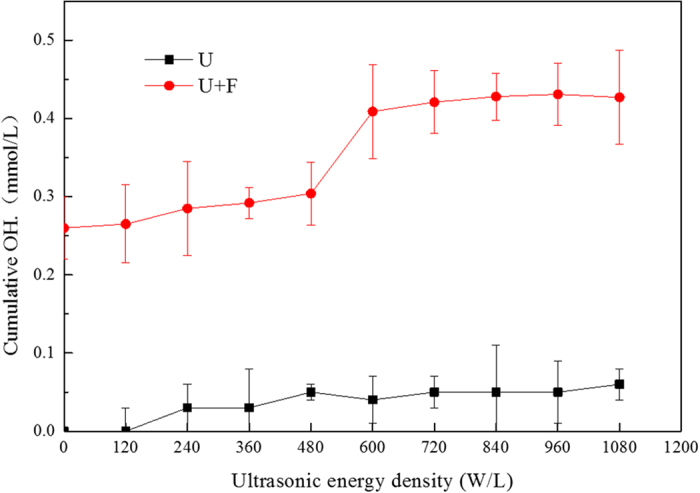
OH• concentration detected using a fluorescence spectrophotometer. Treatment time was 20 min, Fe^2+^ concentration was 0.4 g/L, and H_2_O_2_ concentration was 0.5 g/L.

Sludge pH was measured using a pH meter (Thermo Electron, DRION STAR A214). COD and SCOD were measured using the titration method after digestion. TOC was measured using a TOC analyzer (Shimadzu, TOC-VCPH). EPS was measured using the alkaline extraction method^26^. All data presented in Table 1 and Figs 1, 2, 3, 4, 5 are representative of at least three independent experiments, in which all samples were assayed in triplicate. The OH• concentration was analyzed using the terephthalic acid (TA) trapping protocol^24^. Briefly, the reaction between non-fluorescent TA and OH• produces highly fluorescent hydroxyterephthalic acid (HTA), whose concentration can be determined based on fluorescence measurements and taken as a relative measurement of OH• produced in the solution^24^.

## Results and Discussion

### Organic matter dissolution

#### Time

Carbon is the vector of organic compounds, while the main indices for organic compounds are COD and TOC. As shown in Fig. 1a, during the 0–20-min treatment, the COD contents in the excess sludge supernatants of the F and U+F treatments increased significantly. After 20 min, no further increase was observed. By comparison, the COD of the ultrasound treatment group was negligible. During the 0–20-min F pre-treatment, sludge COD release increased significantly from 292.7 to 510.2 mg/L (0.74-fold) and from 292.7 to 709.0 mg/L (1.4-fold) in the U+F group.

As shown in Fig. 1, after U pre-treatment alone, the release of sludge TOC was negligible. For single Fenton oxidation pre-treatment, during the first 20 min, the TOC concentration increased rapidly from 89.3 to 172.1 mg/L (0.9-fold). No increase was observed after 20 min. In the U+F group, sludge TOC concentration showed linear growth in the first 20 min from 89.3 to 261.5 mg/L (1.9-fold), but showed no further increase after 20 min. The above data demonstrated that an U treatment duration of 20 min was optimal for the dissolution of organic matter and release of carbon elements from excess sludge.

#### Ultrasonic energy density

The effects of increasing ultrasonic energy densities on the release of sludge COD and TOC are shown in Fig. 2.

In the U pre-treatment alone group, sludge COD and TOC did not increase significantly with increasing ultrasonic energy density. However, the SCOD of sludge treated with U+F showed minor increases at ultrasonic energy densities below 240 W/L. At ultrasonic energy densities of 240–720 W/L, SCOD increased rapidly. In addition, no obvious increase was observed at ultrasonic energy densities greater than 720 W/L. At a density of 720 W/L, SCOD increased from 510.2 to 709.0 mg/L (0.4-fold). For TOC, ultrasonic pre-treatment alone did not increase the excess sludge TOC concentration. For U+F with an ultrasonic energy density of 0–720 W/L, the TOC increased from 192.1 to 261.5 mg/L (0.4-fold). Therefore, an ultrasonic energy density of 720 W/L was optimal for the release of organic matter from excess sludge.

COD reflects the amount of reducing substances in water, including organic matter, nitrites, sulfides and ferrous salts, among which organic compounds take the largest proportion. Therefore, COD is used as an index of organic matter concentration in water. The data obtained revealed a strong correlation between the COD and TOC of sludge supernatant. The value of TOC multiplied by 32 was similar to but smaller than COD multiplied by 12 because COD represents both organic matter and reducing substances such as sulfides and amines, while TOC reflects mainly the amount of organic matter. Generally, both the COD and TOC values of sludge supernatant are used as indices of the release of sludge carbon and organic matter.

#### EPS dissolution

Extracellular polymeric substances (EPS) are high-molecular-weight compounds secreted by microorganisms into the environment^27^, including proteins, polysaccharides, humic substances, deoxyribonucleic acids (DNA), lipids, and uronic acid^27^,^28^.

As depicted in Fig. 3, U+F treatment released more EPS from sludge than ultrasound treatment alone. At ultrasonic energy densities of 0 to 600 W/L, the EPS concentration increased from 1038.4 and 1139.7 mg/L to 1068.5 and 1264.3 mg/L for the U and U+F treatments, respectively. EPS did not increase further at ultrasonic energy densities greater than 600 W/L. F treatment was more effective than U treatment.

At an ultrasonic energy density of <240 W/L, the effect of U and U+F treatment on the amount of EPS dissolved from excess sludge was not significant. At ultrasonic energy densities of 360 W/L for the U and U+F treatments, the amount of EPS released increased significantly. Based on these results, releasing EPS from cells of sludge requires higher ultrasonic energy than the release of COD and TOC. EPS, a major component of activated sludge^29^, plays an important role in biological wastewater treatment^30^.

#### Microstructure based on fluorescence microscopy

As shown in Fig. 4, the black flocculent structure of untreated sludge was intact, well-formed and connected (Fig. 4a). The sludge became slightly loose after U treatment for 20 min (Fig. 4b). The sludge became looser after F treatment for 20 min and the large flocculent structure was destroyed (Fig. 4c). Sludge treated by U+F for 20 min resulted in a considerably thinner and looser microstructure than that caused by F treatment (Fig. 4d).

#### Generation of OH•

OH• has the second-most powerful oxidative ability next to fluorine and can non-selectively oxidize the majority of organic contaminants to CO_2_, H_2_O and mineral salts through the fast chain reaction^31^. Because of the extremely high reactivity and short lifetime of OH•, the direct measurement of OH• in aqueous solution remains challenging. Herein, we adopted fluorescence spectrophotometer method to detect the concentration of OH•. The OH• trapped by terephthalic acid (TA) and detect using a fluorescence spectrophotometer^24^. TA is a well-known OH• scavenger and has been extensively used in advanced oxidation systems, particularly sonochemical and radiolysis studies to measure OH• in aqueous solutions^24^.

**Figure 6:**
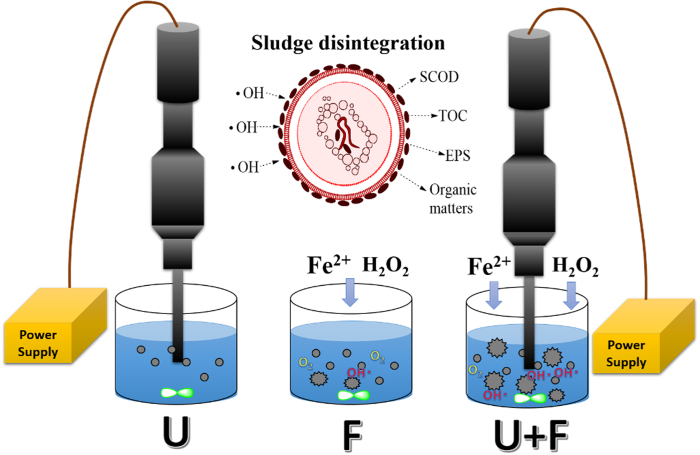
Experimental system of U, F, and U+F (U refers to ultrasound treatment, F refers to Fenton oxidation treatment, U+F refers to ultrasonic coupled to Fenton oxidation)

As shown in Fig. 5, the concentration of OH• detected after U treatment was minimal and could be neglected. F treatment produced 0.26 mM of OH• as the zero point of U+F treatment. When the ultrasonic energy density was 0–480 W/L, the OH• contents of U+F treatment increased slowly. When the density was 480–720 W/L, the OH• contents increased rapidly. The OH• contents of supernatants remained unchanged when the density exceeded 720 W/L for U+F treatment. It is likely that the coupling effect become saturated. Thus, further increases in ultrasound density would have little effect on the amount of OH• produced for U+F treatment. In addition, we adopted another methods to detect OH•. The signal of OH• was detected by ESR Spectrometer^25^ as shown in supplementary information file. The highest OH• signal intensity increased from 568.7 by F treatment to 1106.3 by U+F treatment based on electron spin resonance (Figure S1 and the schematic diagram of our experiment is Fig. 6).

OH• is the primary product of the Fenton reaction and has sufficient oxidation potential to oxidize sludge organic matter, produce organic radicals, and degrade the structure of sludge flocs^32^, which leads to the release and disintegration of organic compounds. Ultrasound creates squirt flows at high temperatures, pressures and speeds, inducing cavity effects^33^,^34^ and effective sludge disintegration^13^,^35^. Ultrasonication or Fenton oxidation alone can induce sludge disintegration. Combined, ultrasound enhances the diffusion of Fenton reagent and thus its utilization rate, ensuring thorough contact between OH• and organic matter, which improves the efficiency of sludge disintegration. Ultrasound has also been reported to produce OH•^26^ and Fenton reagent is known to generate massive microbubbles rich in oxygen, promoting the cavity effect of ultrasound and the generation of OH•. U+F treatment resulted in increased levels of OH• than compared to U or F treatment alone, supporting our hypothesis. U+F treatment significantly increases the amount of OH• and enhances the degradation of organic compounds, thus releasing more COD, TOC, and EPS from sludge (Figs 1, 2, 3). The release of COD and TOC represents the dissolution of organic matter. The release of organic matter and disintegration of sludge were promoted by U+F treatment according to the qualitative and quantitative analysis of OH•.

## Additional Information

**How to cite this article**: Gong, C. *et al.* Ultrasonic application to boost hydroxyl radical formation during Fenton oxidation and release organic matter from sludge. *Sci. Rep.* **5**, 11419; doi: 10.1038/srep11419 (2015).

## Supplementary Material

Supplementary Information

## Figures and Tables

**Table 1:** Characteristics of the excess sludge analyzed in this study.

**No.**	**pH**	**Moisture**	**COD**	**TOC**	**TC**	**N**	**P**
Units		Content%	mg O_2_/L	mg/L	mg/L	mg/L	mg/L
Mean	6.82	98.8	17868.4	89.3	117.7	98.8	268.8
SE	0.06	0.2	697.5	8.6	8.5	12.7	36.9
