# Supplementary material for: Ultrasonic application to boost hydroxyl radical formation during Fenton oxidation and release organic matter from sludge
Source: Sci Rep. 2015 Jun 12;5:11419. doi: 10.1038/srep11419 (PMC4464359; doi:10.1038/srep11419)
Supplement: Supplementary Information [file srep11419-s1.pdf]

Ultrasonic application to boost hydroxyl radical formation during Fenton oxidation and release organic matter from sludge

Changxiu Gong [a], Jianguo Jiang [a,b,c,*], De'an Li [a], Sicong Tian [a]

a School of Environment, Tsinghua University, Beijing 100084, China

b Key Laboratory for Solid Waste Management and Environment Safety, Ministry of Education of China, China

c Collaborative Innovation Center for Regional Environmental Quality, Tsinghua University, Beijing, China

* Correspondence to jianguoj@mail.tsinghua.edu.cn

Determination of hydroxyl radicals

The hydroxyl radicals were trapped using 5,5-dimethyl-1-pyrroline-N-oxide and detected using an ESR Spectrometer (JEOL Company, JES-FA200).[23] Typical spectrometry parameters were as follows: center field of 323.3 mT, sweep width of +/- 5/1 mT, sweep time of 2.0 min, microwave frequency of 9056.1 MHz, power of 0.998 mW and modulation frequency of 100 kHz.

OH• intensity was detected using an ESR Spectrometer:[23] A total of 2 mL of 0.1 mol/L DMPO was added to a 10-mL beaker, after which 2 mL of sludge solution and 1 mL of 0.01 M hydrochloric acid were added. The system was then treated with 720 W/L ultrasonication for 6 s, followed by addition of 4 mL of high-purity water or 2 mL of 0.4 g/L Fe^{2+} solution and 2 mL of 0.50 g/L H_2O_2 solution, ensuring identical final volumes and DMPO and sludge concentrations. Sampling was performed with a capillary, the bottom of which was sealed with plasticine after sampling. The samples were placed in the resonant cavity of an electron spin resonance (ESR) spectrometer. The above procedures were completed within 2 min. The assay was then started followed by analysis for 2 min.

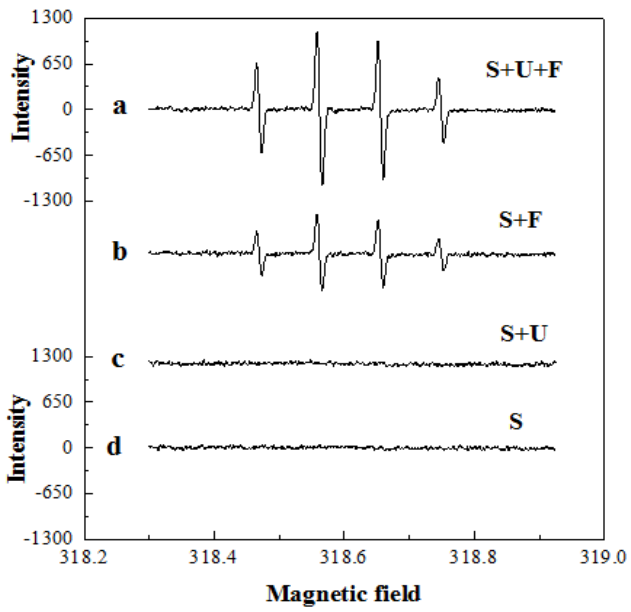

Figure S1. OH• intensity detected using an ESR Spectrometer. a: U+F treatment; b: F treatment; c: U treatment; d: No treatment. The ultrasonic energy density was 720 W/L; the concentration of Fe^{2+} was 0.4 g/L and H_2O_2 was 0.5 g/L; treatment time was 6 s.

As shown in Figure S1, no OH• was detected in raw sludge using DMPO, a capture agent. The same result was found for sludge pre-treated with ultrasonication. Under F treatment, the highest OH• signal intensity was 568.7. Under U+F treatment, this value reached 1106.3, representing a 1.9-fold increase. Therefore, the OH• concentration increased significantly after ultrasonication.

Table S1. Characteristics of the supernatant from excess sludge analysed in this study.

No.	Fe	Ammonia	Sulphides	Nitrites
Units	mg/L	mg/L	mg/L	mg/L
Mean	8.4	412	17.5	0.5
SE	0.7	24.2	2.5	0.05

Fe: detected by Inductively coupled plasma (IRIS Intrepid Ⅱ XSP, Thermo company).

Ammonia: detected by Ammonia Medium Range (HI96715, HANNA company)

Sulphides: Soluble sulphides detected use indirect method by Inductively coupled plasma (IRIS Intrepid Ⅱ XSP, Thermo company).

Nitrites: The Determination Methods of Nitrite and Nitrate in Food, China National Standards (GB/T 5009.)
